# Allegheny County Medical Society

**Published:** 1892-09

**Authors:** 


### *SOCIETY REPORT.*

### ALLEGHENY COUNTY MEDICAL SOCIETY.

PITTSBURG, PA., June 10, 1892.

DR. CHAS. S. SHAW read a paper on the

ARTIFICIAL FEEDING OF INFANTS. (See page 389.)

Following is the discussion of the same:

DR. SMALL: Three years ago I commenced this system of feeding children. I followed as nearly as I could the mode of sterilization as given by Dr. Rotch, to which Dr. Shaw has referred, and I used also the mixture. It agreed much better with the children than any other food, and since then I have used it more or less in my practice. Generally, the patients I have had have been such that it was necessary to have the apparatus as cheap as possible. I had a case a year ago, of a child six or seven weeks old when brought to me. I did not attend the birth. The mother had puerperal fever, so could not nurse the child at all. A "steamer" for cooking, or for keeping food warm, made an admirable sterilizer. Recently I had a boy who could not take his mother's milk, and I had the father have a sterilizer made. He bought a tin bucket about ten or twelve inches high, and had a platform made in it three inches from bottom, and fitted it with a close cover. It worked very well.

DR. DAVIS: I feel that the subject before us to-night is of great importance. I believe there is not one thing that a physician is called upon to do that perplexes him more than that of substituting artificial food for the mother's food, for the reason that the very things that work very successfully with one child utterly fail with another. While it is true to say, as we have had it in the paper to-night, that sterilized milk is a good substitute for mother's milk, yet there are some cases in which it is actually poison. There are some children to whom all milk, in whatever form, seems poison, and acts the same as if they had taken poison instead of milk. I think it is the experience of every one, that in these cases you have to take them off of milk entirely, keeping them off for several days to a week or more, and even then when resuming milk, you find that it still acts badly, and have to desist for a few days longer. They can then return to the milk which seemed poisonous, and digest it well.

There is one point in Dr. Shaw's paper that I must beg leave to differ with—the summary manner in which he disposes of all prepared food. I know there are a great many bad foods in the market. There are a great many, I might say all of them, which make greater claims than they are able to fulfill, but to dismiss them all with a wave of the hand and say there is no good in any of them is rather contrary to the experience of many of us. I have seen children suffering all the terrors of bad feeding put on some of these foods and grow right on to vigor and health, and therefore to say they are absolutely no good is saying too much. Now, as I understand prepared foods, they can be classed in three classes: The milk foods, the grain foods and the meat foods, and I would not stop two minutes to distinguish between the foods in these classes: The grain and the meat foods we have nothing to do with in children under three months of age. After a child is able to take care of starchy matter we advance to grain food, and when teeth appear we advance to meat food.

The truth of the matter is, we find that milk taken directly from a cow, and almost directly put into thoroughly cleansed bottles and corked up, without more chance of infection than would occur between a child's mouth and a mother's breast will often disagree. On the other hand, we have taken children off the sterilized milk and put them on cow's milk, which suited them without any sterilization or preparation whatever, and it agreed perfectly. Now, I believe it is not so much the trouble with bacteria; I believe it is chemical changes. I think our milk collected through Ohio, Western Pennsylvania and West Virginia, milked the night before, kept possibly in imperfect spring-houses, put in cans and transported over long distances on our railroads, brought to the city and dished out again and hauled out over rough streets on hot days, cannot be a good food. I believe, even if it is bottled and sterilized, after going through these changes, it is not fit food, and I am reaping more success with the condensed milk than I am with any form or shape of the milk brought to this city, some of which is reported as being specially sterilized, and brought in glass bottles, etc.

DR. KŒNIG: It seems to me the reader of the paper has overlooked one point in bringing this subject before us. He has attributed all the trouble in the digestion of milk to the milk itself, and has laid no blame at all on the stomach. One would expect that nature would provide infants' stomachs with the necessary ferment to act upon the milk, so as to render it suitable for absorption, but I believe this is frequently not the case, and that by the substitution of a ferment obtained from some other animal, we can render that milk fit for absorption. I believe I have seen the life of a child saved by the administration of pepsin with its milk.

DR. GREEN: I am satisfied that every physician who has been so unfortunate as to engage in the treatment of children brought up on these diets has a task before him. I have in numerous cases resorted to almost any and every method that I could find, preparing different foods in different manners, and am sorry to announce in many instances utterly failed in the end, but I have been successful very often in the feeding of a child on artificial diet, and I feel free to state while I am not wedded to any particular food or any particular manner of preparing food, I believe the condensed milk is very good and possibly proves successful in a larger number of cases than any other single milk diet that is artificially applied or given. I agree with Dr. Davis; I think it is well to examine the cow as well as the baby and food. I think, without looking to all these factors in the case, we many times fail, and if we fail to find a sufficient quality of food, and if we fail to find proper digestion of the food given to the child, by examining the different points or factors in the case, we often can arrive at the preparation of a particular food for a particular case. I think Dr. Kœnig's remarks were well placed. I feel that many times it is not the food; it is the stomach.

DR. LANGE: There seems to be a little diversity in the argument. The title of Dr. Shaw's paper was infant feeding, and although that is very hard to consider without considering some of the diseases, still it seems the discussion of diseases of infancy has got the upper hand. No one will claim that any food will prevent cholera infantum, "summer complaint"; that if we had an ideal food that summer complaint would be banished from the land. The conditions of the stomach play the most important part and not the nurse. It has happened to me in my time, to have three infants who, we became convinced, could take absolutely nothing into the stomach; they probably could not have taken the ideal food if we had had it. These three infants were deprived of all food and were only annointed, one for a period of three weeks, with almond oil, oil of sweet almond, and the infants received nothing into the stomach but a little water and recovered and grew strong under that treatment, afterwards taking the milk well, which formerly disagreed. Now the point I desire to make is, food, even if it is the best, is not always acceptable to infants. Then, as I understand it, shipping the milk, shaking the milk, driving the milk in the sun and canning the milk, can do it no harm if it is sterilized. It is not the heat that spoils it, but the bacteria that are in it. So if it were sterilized, it might be shipped around the world and come back as good as it was. That is a strong argument for sterilization, but we should have a standard of the best infant's food, irrespective of peculiarities, irrespective of differences that may exist between mothers and cows. The same differences generally exist in every piece of meat that we all eat. Further, it has been said that it would be beneficial to add a digestive ferment, as for instance, pepsin. Now pepsin is getting to be old, and I think the majority of you will agree with me if you studied it as close as I have, that it is a substance which is entirely useless. I do not believe a physician ever got any result from it, unless he had used along with it the important factor, hydrochloric acid, and I can promise every gentleman, in cases of gastric disturbances,if he will use pepsin without hydrochloric acid, and then use the acid without pepsin, the last will bear much better fruit than the first.

DR. SHAW: It is a fact that some children will thrive on almost any food; a fortunate fact. I know of children that from a very early age, two or three months, have been fed almost exclusively on boiled barley. It has been often said that infants would not digest starch, but sometimes they will, though it is evident that starch is not as good a food for children as milk. The probable reason for the digestion of starch in the absence of a proper amyloid digestive apparatus is the mucus of the stomach and the intestines. The suggestion of one gentleman as to the time of putting the lime water in the milk I do not think calls for any particular discussion. It is to my mind only a matter of convenience. It is only important that it be put in. Infant's foods on the market, in my experience, have not been satisfactory. Those of them that are used with most satisfaction are used with milk, and the milk that is added to them, and the boiling that is usually directed to be done when making the mixture, makes a good food, because it is sterilized milk. Some of them have elements of nutrition that are suitable to the digestive capacities of an infant, but many have not. I have had a very annoying but instructive experience recently in the Rosalia Foundling Asylum. We have had constantly in charge from fifty to sixty infants under ten months old. About one-half of these children were fed on bottled food. The opportunity was excellent for the trying of a great many artificial foods, and we tried carefully, and with the help of skilled nurses and assistants, almost all the foods in the market, and none of them were satisfactory. The sterilized milk as prepared by Dr. Rotch's formula certainly suited these children best. I am satisfied that where it fails in a healthy infant the cause will be that it is not properly prepared and sterilized.

